# Can follow up lung ultrasound in Coronavirus Disease-19 patients indicate clinical outcome?

**DOI:** 10.1371/journal.pone.0256359

**Published:** 2021-08-25

**Authors:** Tatjana Hoffmann, Peter Bulla, Lisa Jödicke, Constantin Klein, Sarah M. Bott, Ronald Keller, Nisar Malek, Eckhart Fröhlich, Siri Göpel, Gunnar Blumenstock, Stefano Fusco

**Affiliations:** 1 Department of Internal Medicine I, Section of Gastroenterology, Gastrointestinal Oncology, Hepatology, Infectiology and Geriatry, University Hospital Tübingen, Tübingen, Germany; 2 Department of Clinical Epidemiology, Eberhard-Karls-University, Tübingen, Germany; Kaohsuing Medical University Hospital, TAIWAN

## Abstract

**Purpose:**

To evaluate whether there is a change in findings of coronavirus disease 2019 patients in follow up lung ultrasound and to determine whether these findings can predict the development of severe disease.

**Materials and methods:**

In this prospective monocentric study COVID-19 patients had standardized lung ultrasound (12 area evaluation) at day 1, 3 and 5. The primary end point was detection of pathologies and their change over time. The secondary end point was relationship between change in sonographic results and clinical outcome. Clinical outcome was assessed on development of severe disease defined as need for intensive care unit.

**Results:**

Data of 30 patients were analyzed, 26 patients with follow-up lung ultrasound. All of them showed lung pathologies with dynamic patterns. 26,7% developed severe disease tending to have an ubiquitous lung involvement in lung ultrasound. In patients with need for intensive care unit a previously developed increase in B-lines, subpleural consolidations and pleural line irregularities was more common. A statistically significant association between change in B-lines as well as change in pleural line irregularities and development of severe disease was observed (p<0,01).

**Conclusion:**

The present study demonstrates that follow up lung ultrasound can be a powerful tool to track the evolution of disease and suggests that lung ultrasound is able to indicate an impending development of severe disease in COVID-19 patients.

## Introduction

Up to 1st March 2021, there have been 113,820,168 officially confirmed cases of severe acute respiratory syndrome coronavirus type 2 (SARS-CoV-2) infections with a mortality of 1,6% in Germany and 2,3% worldwide [1, 2]. Coronavirus disease 2019 (COVID-19) typically presents with systemic and respiratory manifestations. About 20% of hospitalized patients develop severe disease, mostly defined as fatal outcome or need for intensive care unit (ICU) [3–6].

The SARS-CoV-2 affects its cellular entry via attachment of its virion spike protein to the angiotensin-converting enzyme 2 receptor, commonly found on alveolar cells of the lung epithelium and on several other tissues, underlining the development of alveolar damage and progressive respiratory failure [7].

Experts have recommended computed radiography (CT) for evaluation of COVID-19 [8, 9], but the high contagiousness of SARS-CoV-2 and the risk of transporting unstable patients makes CT a limited option. Recent studies confirmed that lung ultrasound (LUS) has an accuracy similar to that of CT in detecting lung abnormalities [10] and that use of LUS helped in avoiding serial chest x-rays and CT scans in COVID-19 patients [11].

The advantages of LUS in terms of portability, safety and possibility of repeating the examinations without exposure to radiation cannot be overlooked. Further, LUS can produce real-time and dynamic images [12].

Already during the 2019–2020 pandemic non-specific and characteristical sonographic findings were described, tending to have a bilateral, peripheral and posterobasal predominance [3, 13–15]: irregular pleural line with multiple B-lines, subpleural consolidations and restitution of aeration during recovery. The predominant pattern is of varying degree which seems to be correlated with the severity of the lung injury [14].

This is why former studies already recommended sonographic monitoring [14, 16]. To our best knowledge, prospective studies collecting data of follow up LUS in COVID-19 patients are still missing.

With this study we aim to find out whether follow up LUS allows indicating an impending development of severe disease by providing prognostic information in COVID-19 patients.

## Materials and methods

### Study design

This prospective and monocentric diagnostic study was approved by the local Ethics Committee at the medical faculty in Tübingen with a written consent 04/2020 (project number 230/2020B02). LUS data were evaluated by two experienced sonographeurs (DEGUM II, DEGUM III) who were blinded to each other and to clinical, radiological and blood data. Between March 2020 and September 2020, a total of 30 patients fulfilled the inclusion criteria (age > 18 years, positive SARS-CoV-2 PCR assay, in-patient care and ability to sign our written consent) and were selected as participants. Exclusion criteria were a negative SARS-CoV-2 PCR assay or a prescribed heart failure.

### Study schedule

LUS was performed on day 1 (within 48 hours since the first presentation in hospital), day 3 and day 5, provided patients were still on the isolation ward. On the days of LUS, oxygen demand as binary variable (yes/no) was documented. In addition, ICU treatment during the hospitalization for any reason was documented.

### LUS data collection

**Fig 1:**
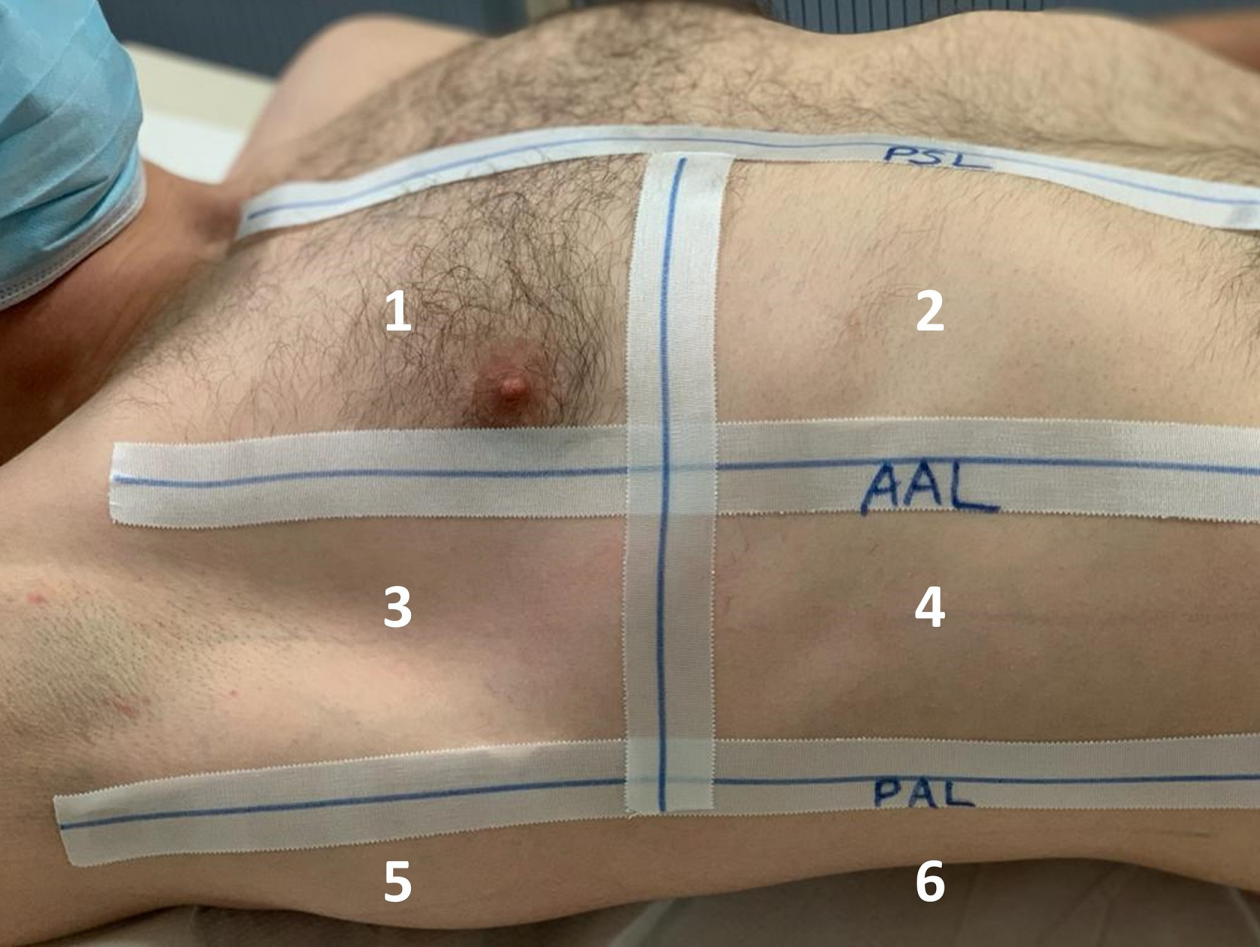
Lung areas for the right lung, PSL = parasternal line, AAL/PAL = anterior/posterior axillary line.

LUS studies were performed at bed-side by trained physicians, if possible as part of the daily rounds. Exams were performed with Philipps Sparq. Transducer was placed longitudinal and each intercostal space of upper and lower parts of the anterior, lateral, and posterior regions of the left and left chest wall was scanned (Fig 1). Videos of all 12 lung areas were scored. We used the convex transducer C6-2 and for given regions we added the linear transducer L12-4 for more accurate assessment of the pleura. Images were documented as standardized pattern in PACS, visualization software for imaging. All data were gained from entirely computerized medical records.

### LUS analysis

**Fig 2:**
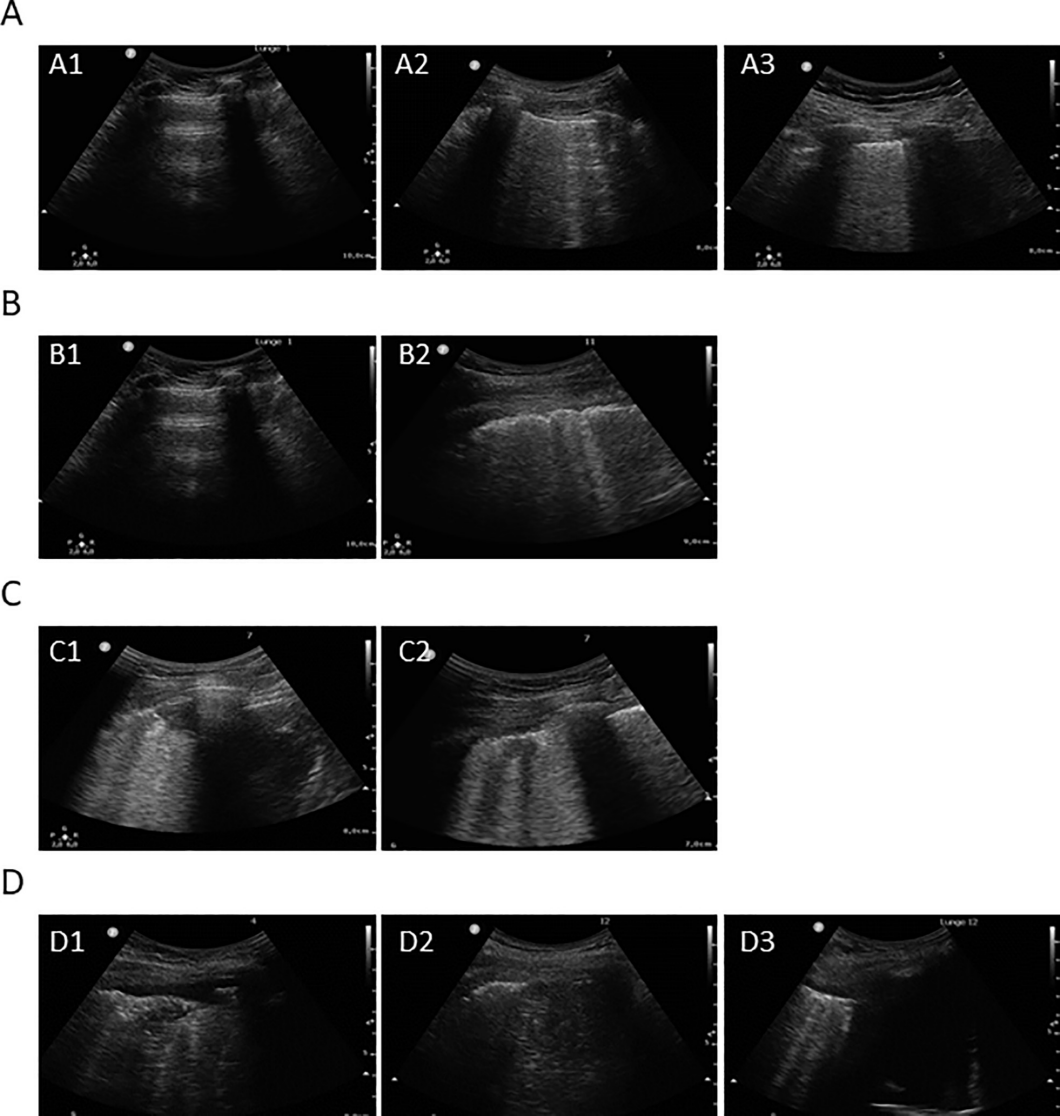
Normal aeration with horizontal A-lines (A1), loss of aeration with well-defined B-lines (A2) and coalescent B-lines (A3); normal (B1) und irregular (B2) pleural line; subpleural consolidations (C1 + C2); larger consolidations with air bronchogram (D1 + D2) and pleural effusion (D3).

Videos of all 12 lung areas were evaluated individually by two experienced sonographeurs. The worst affected areas with obvious pathologies were selected as regions of interest (ROIs), which could be one or more of the 12 lung areas. The following features (Fig 2) were evaluated in the whole lung:

- B-lines: comet-tail artefacts fanning out from the lung-wall interface and spreading up to the edge of the screen. Inspired by a former study [3] we used a scoring system and calculated a cumulative LUS score of the 12 areas. B-lines were divided to B1 (separated B-lines that correspond to moderate lung aeration loss) that was equal to 1 point, and B2 (coalescent B-lines that correspond to severe lung aeration loss) that was equal to 2 points. Thus, a total LUS score of 0 was normal, and 24 was worst
- Pleural line irregularities: incontinuous, unsmooth or irregular pleural line, assessed as binary variable (yes/no)
- Subpleural consolidations: smaller nodules defined as poorly delineated hypoechoic tissue structure, assessed as binary variable (yes/no)
- Complications: pleural effusions or consolidations with occasional air bronchograms, assessed as binary variable (yes/no)

### Statistical analysis

Data on LUS examinations at day 1, 3 and 5 were collected and entered into a MS Excel worksheet. The basic statistical analysis was performed using the JMP 15.2 statistical software (SAS Institute Inc., Cary, NC, USA). All sonographic features in follow up LUS were evaluated with increase, stable or decrease. Categorical data were reported as number and percentages and displayed graphically as bar charts and as mosaic plots for two-way contingency tables [17]. The patient-specific course of the total LUS score is additionally displayed in a line chart. Sensitivity, specifity, and predictive values of LUS features (last LUS-baseline LUS) for need of ICU transfer were calculated. With respect to the restricted number of observations, the exact Fisher-Freeman-Halton test [18] was performed for testing statistical association, using IBM SPSS Statistics 27. Continuous data were summarized with the mean and standard deviation and were compared by use of the two-sample *t* test. *P* values ≤ 0.05 were assumed to reflect statistical significance. Inter-observer variability for LUS analysis was determined by a second independent blinded observer. Inter-observer variability for cumulative B-lines was assessed using the Bland–Altman method. The degree of agreement of categorical ratings between observers in relation to binary variables (pleural line irregularities and subpleural consolidations) was assessed using Cohen’s kappa. Kappa values were reported with 95% confidence intervals.

## Results

Between March and September 2020 30 patients at the isolation ward participated in the study. Patient demographics are represented in Table 1. During the hospital stay, 8 patients had to be transferred to ICU (26,7%). In-hospital mortality was 16,7%.

**Table 1:** Patient demographics and clinical baseline characteristics.

**Age, mean (SD)**	**70 (13.3)**
**Sex, male (%)**	**17 (57%)**
**Transfer to ICU (%)**	**8 (27%)**
**In-hospital mortality (%)**	**5 (16%)**
**Prior history of heart disease (%)**	**12 (40%)**
**Prior history of lung disease (%)**	**11 (37%)**
**Prior history of immunosuppressant therapy (%)**	**3 (10%)**
**Prior history of cancer (%)**	**7 (23%)**
**Initial oxygen supplementation**	**16 (53%)**
**Fever (≥ 38.3°C)**	**8 (27%)**
**Dyspnoea**	**14 (47%)**
**Tachypnoea (≥ 20/min)**	**24 (80%)**
**Oxygen saturation (< 95%)**	**17 (57%)**
**Early warning score ≥ 8**	**14 (47%)**
**Radiological infiltrate(s)**	**19 (63%)**

18 out of 30 patients were examined on all scheduled days. 12 out of 30 were discharged from the ward earlier: 8 out of 12 after two performed LUS, 4 out of 12 without follow up. Most of the discharged patients could be released home due to their improved general condition, two of them had to be transferred to ICU before the third LUS was performed.

**Fig 3:**
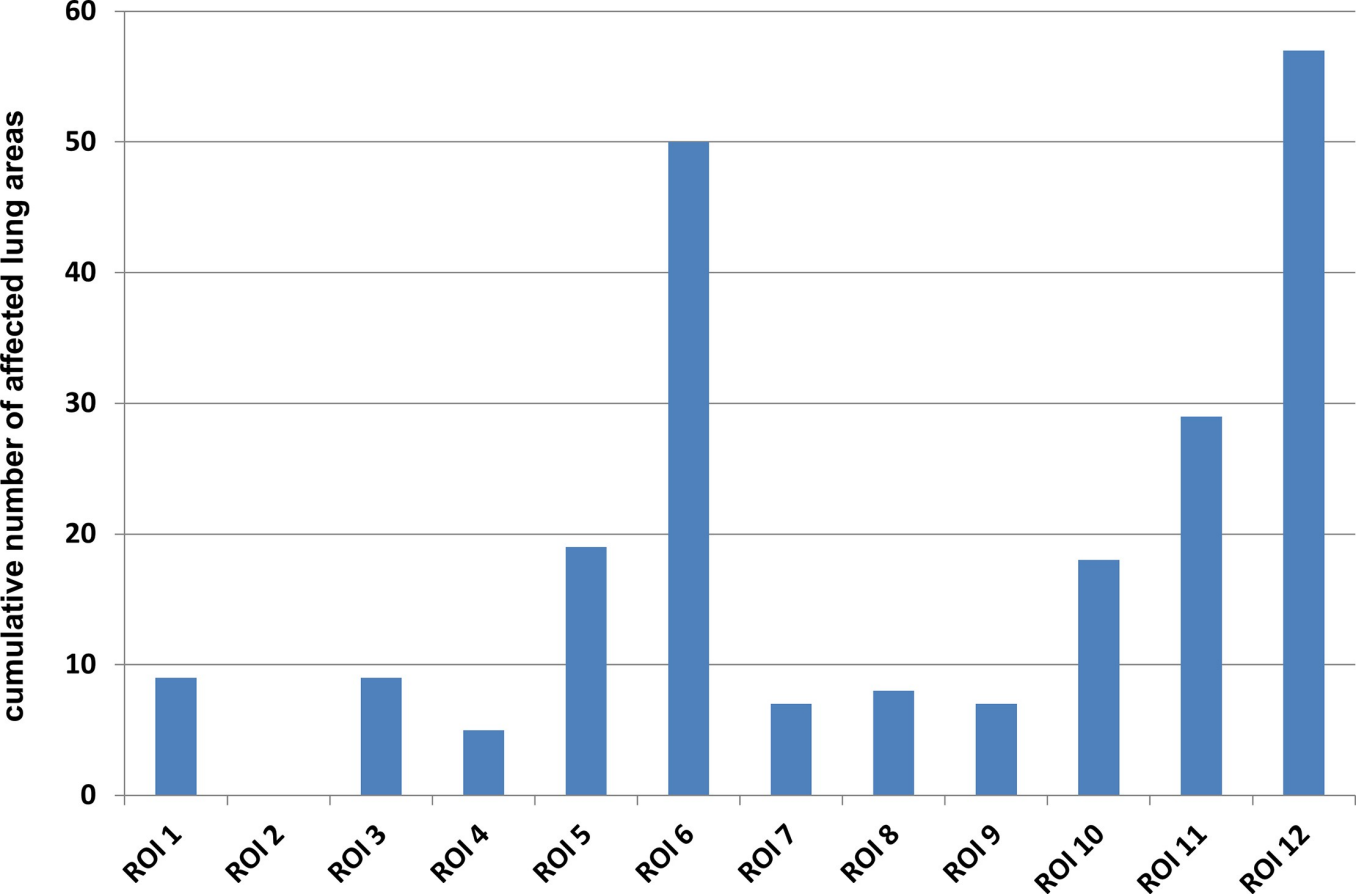
Distribution of ROIs in LUS (d1 + d3 + d5).

The worst affected areas (Fig 3) were the posterior lower fields, followed by the posterior upper fields. There was a tendency towards bilateral lung involvement: regarding the last performed LUS 100% of the patients with transfer to ICU showed bilateral lung involvement, whereas 38,8% of the remaining had unilateral lung pathologies. Affection of upper and anterior areas was observed more frequently in patients with ICU treatment (100%) than in the remaining patients (45,5%).

**Fig 4:**
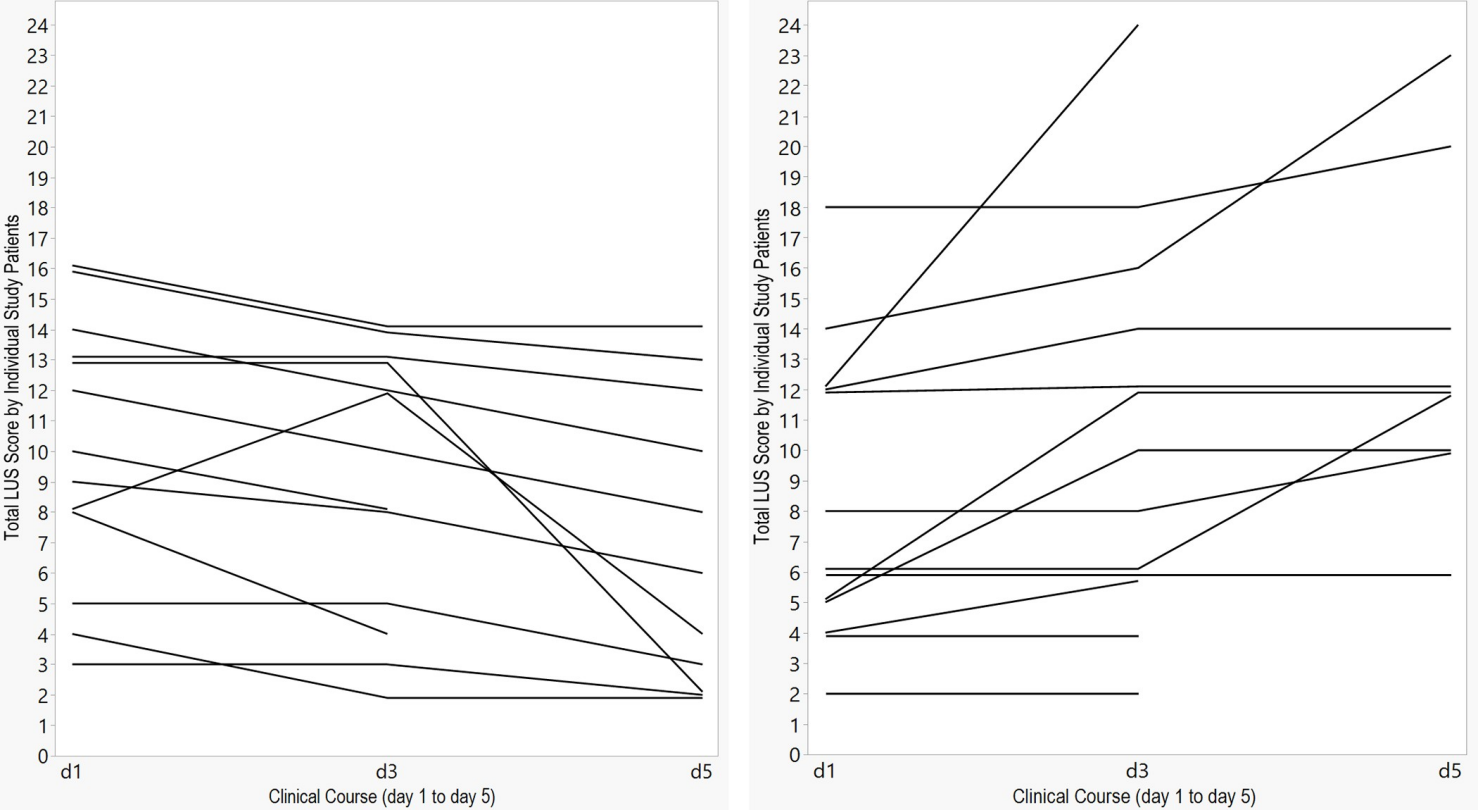
Patients with decrease of cumulative B-lines (total LUS score) on the left side (n = 13) and increase (n = 9) or stable results (n = 4) on the right side (last LUS-baseline LUS).

Inter-observer variability of cumulative B-lines evaluation was assessed using the Bland-Altman method. The 95% range of agreement for the B-lines evaluations was from -1,83 to 2,28. This means, we expected the two observers to give measurements that differ by less than about 2 points in either direction. Thus, cumulative B-lines could be averaged for statistical analysis. Fig 4 gives an overview of the individual courses of cumulative B-lines.

Baseline LUS showed increased B-lines in 100%, pleural line irregularities in 83,3% and subpleural lesions in 70%. The second LUS displayed increased B-lines in 100%, pleural line irregularities in 92,3% and subpleural lesions in 80,7%. The third LUS demonstrated increased B-lines in 100%, pleural line irregularities in 88,8% and subpleural lesions in 83,3%.

**Fig 5:**
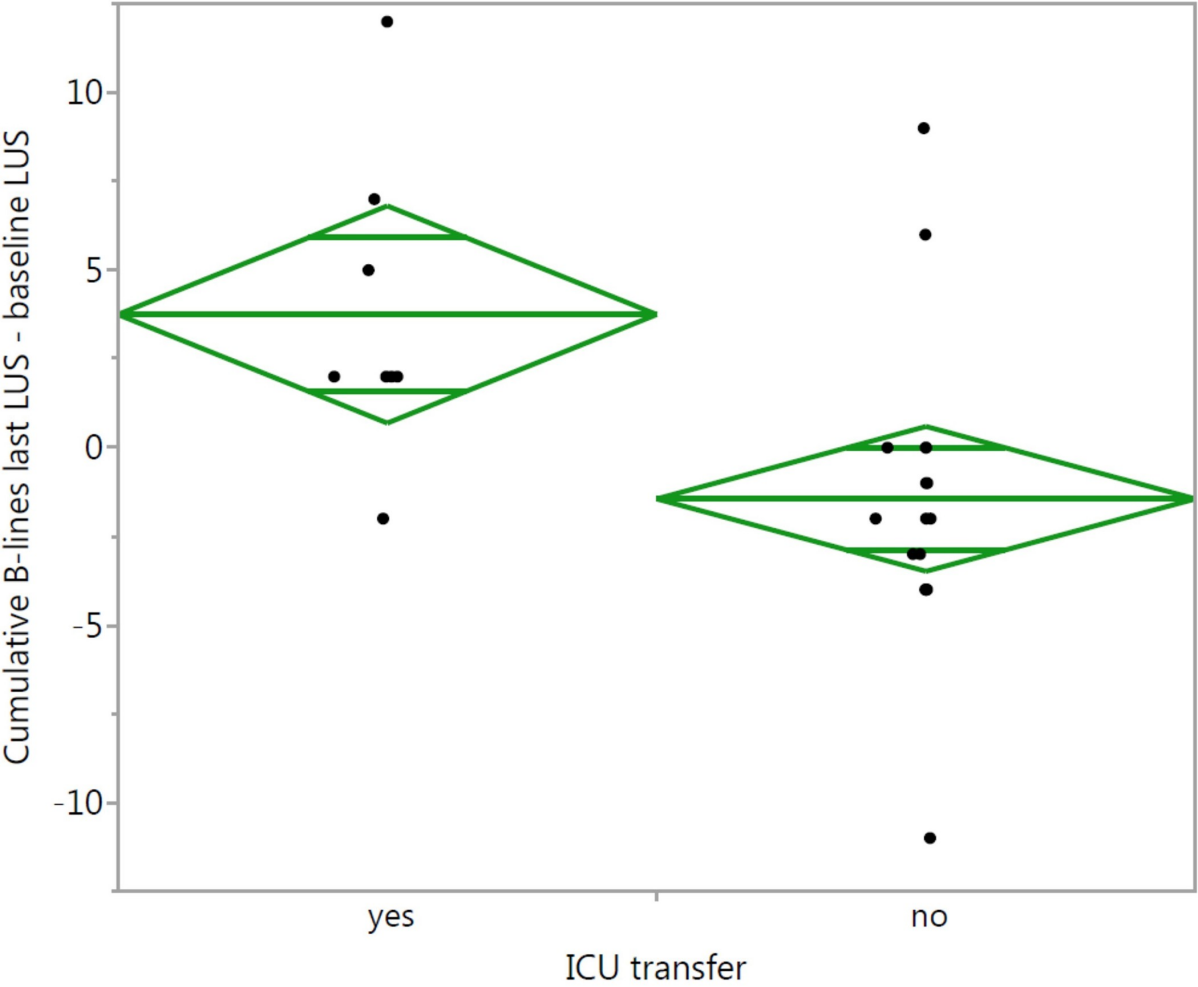
Oneway analysis of cumulative B-lines last LUS—baseline LUS by ICU transfer (two group diagram).

A statistically significant association between change in B-lines and ICU transfer was observed (p = 0,0076). Patients who were transferred to ICU previously developed an increase in B-lines in 87,5% (mean 3,8, standard deviation 4,2), in the remaining group in only 11,1% (mean -1,4, standard deviation 4,2) (Fig 5).

**Fig 6:**
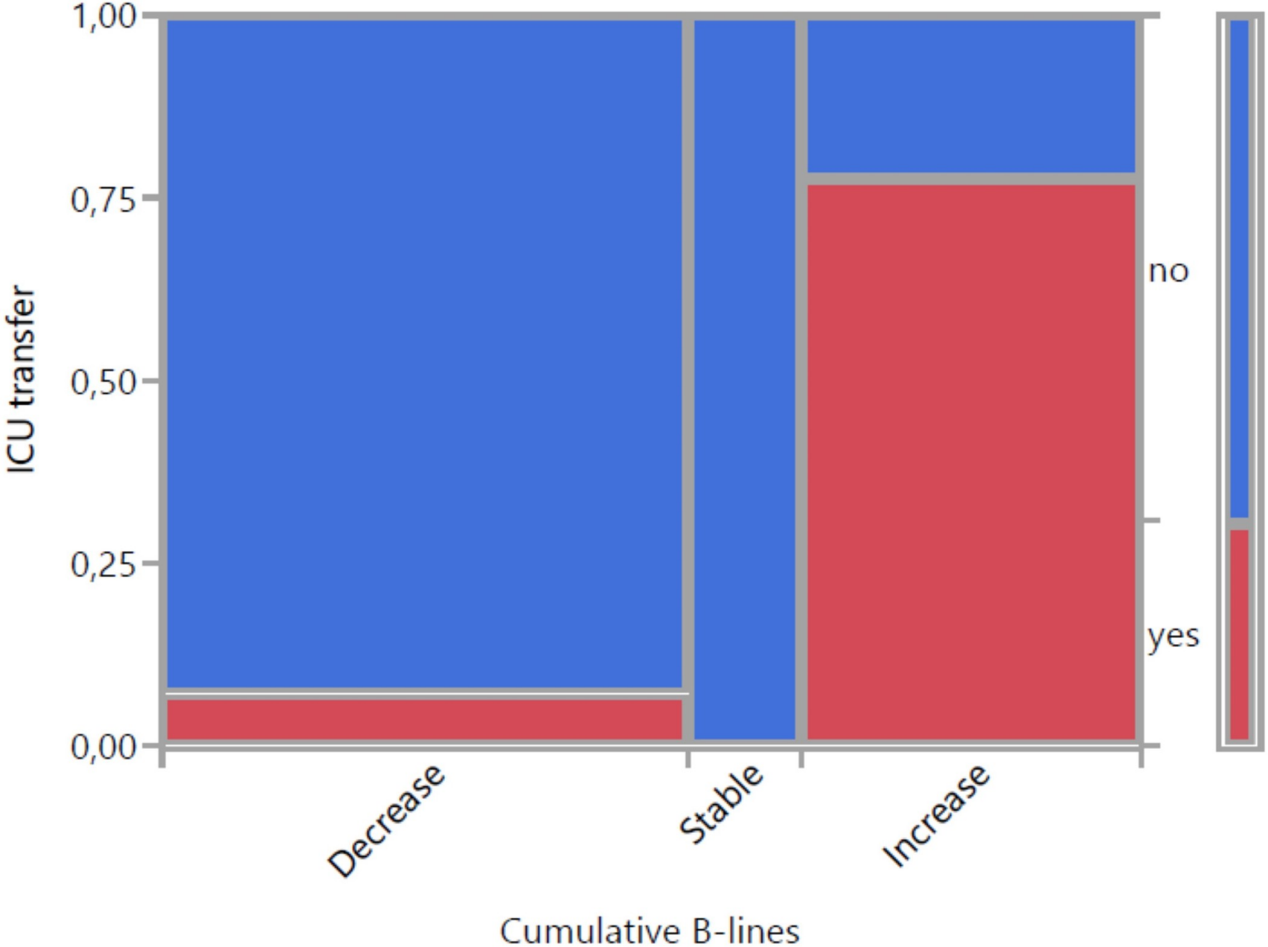
Visualization of change in B-lines with 3 categories and ICU transfer (mosaic plot).

Fig 6 demonstrates the relationship between change in B-lines and ICU transfer. The majority of patients with decrease or stable results did not need ICU treatment correspondingly a negative predictive value of 94%. The positive predictive value of an increase in B-lines for ICU transfer was 78% (7/9).

ICU transferred patients gained cumulative B-lines (last LUS-baseline LUS) in an average of 3,7 points with a mean value of 11,3 points in total LUS score. The remaining patients lost an average of 1,4 points with a mean value of 7,4 points. Sensitivity and specifity of an increase in B-lines for indicating ICU transfer were 88% and 89%, respectively.

**Fig 7:**
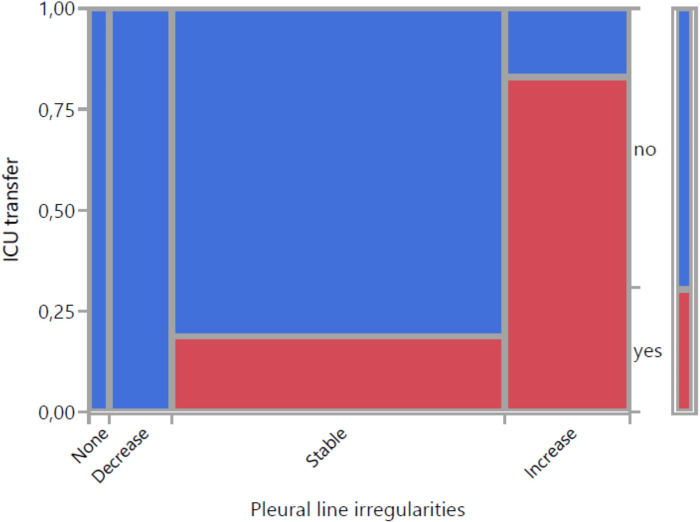
Visualization of change in pleural line irregularities with 4 categories and ICU transfer (mosaic plot).

Fig 7 demonstrates the relationship between change in pleural line irregularities and ICU transfer. 17 out of 20 patients without irregularities or decrease did not need ICU-transfer (negative predictive value of 85%). Of those with an increase 5 out of 6 had to be transferred to ICU (positive predictive value of 83%). Patients with ICU transfer previously developed an increase in 62,5%, the remaining patients in only 5,6%. Sensitivity and specifity of an increase in pleural line irregularities for indicating ICU transfer were 63% and 94%. The association of both variables was statistically significant (p = 0,011). With a confidence interval of 0.81 (0.66–0.97) strength of agreement between the two observers in relation to pleural line irregularities was high.

**Fig 8:**
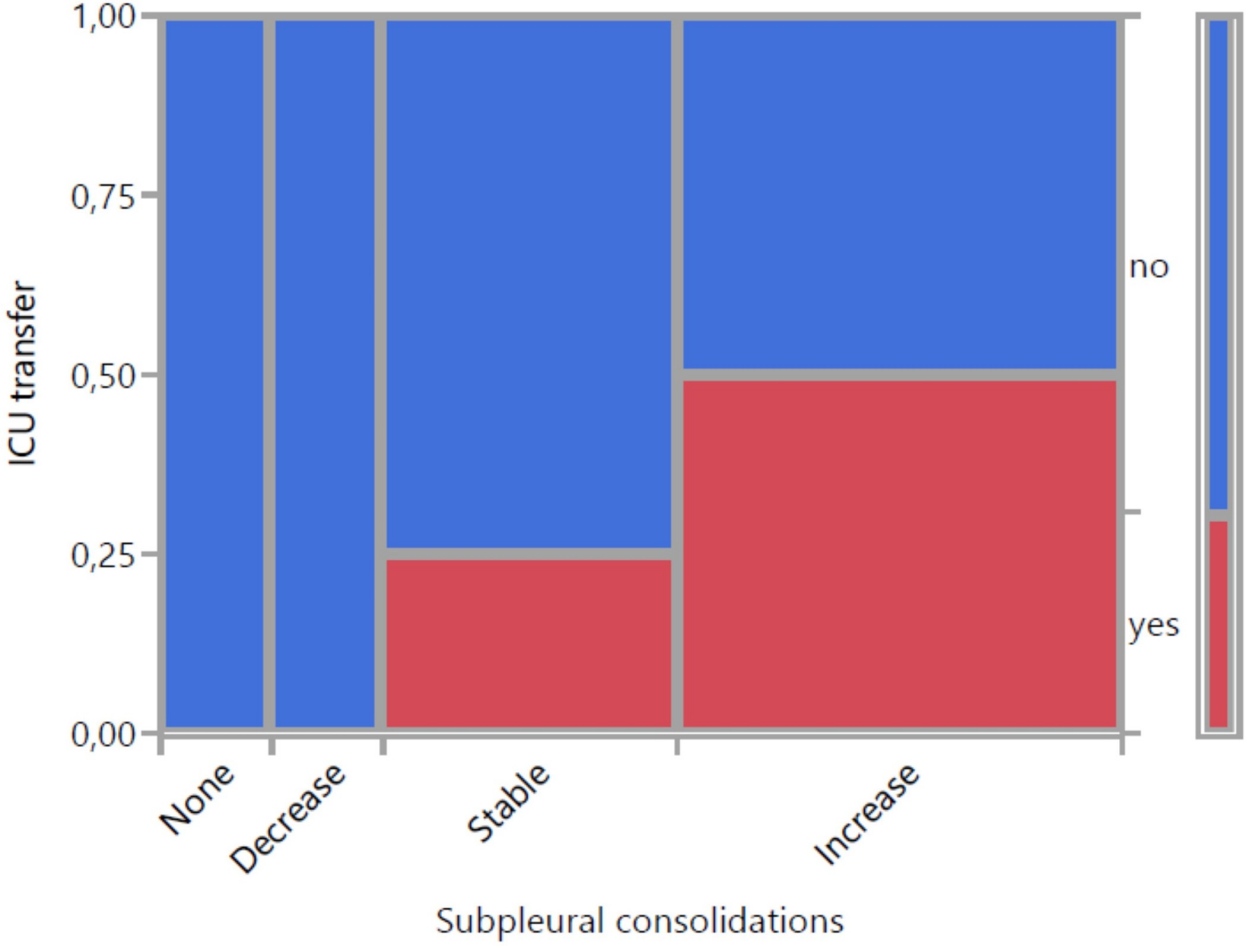
Visualization of change in subpleural consolidations with 4 categories and ICU transfer (mosaic plot).

Fig 8 visualizes the change in subpleural consolidations and ICU transfer. 6 out of 12 patients with an increase had to be transferred to ICU. That corresponds to a positive predictive value of 50%, the negative predictive value was 86%. ICU transferred patients had previously developed an increase in 75%, the remaining in only 33,3%. Sensitivity and specifity of an increase in subpleural consolidations for ICU transfer were 75% and 67%. However, a statistically significant association could not be evidenced (p = 0,225). With a confidence interval of 0.93 (0.86–1.0) strength of agreement between the two observers in relation to subpleural consolidations was high.

Development of larger pulmonary consolidations (5/30) was rarely seen, but more frequently in patients with ICU-transfer (4/8) than in the remaining (1/22). Pleural effusions (5/30) were also rare and observed more frequently in patients with ICU treatment (2/8) than in the patients without ICU treatment (2/22). Empyema and pneumothorax were not observed.

**Fig 9:**
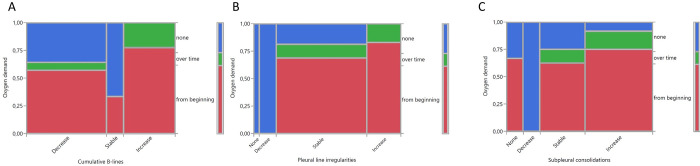
Association between change in cumulative B-lines (A), pleural line irregularities (B), subpleural consolidations (C) and oxygen demand.

There was no statistically significant association between sonographic features and oxygen demand. Nevertheless, one can determine that an increase in B-lines and in subpleural consolidations was always associated with oxygen demand from beginning on or over time, an increase in subpleural consolidations in at least 91,6% (Fig 9).

## Discussion

Despite major efforts to contain the infection, COVID-19 is still a worldwide pandemic. In individual patients, the clinical course is highly dynamic with progress to acute respiratory distress syndrome [19]. There is a need for rapid assessment of the severity and to track the evolution of disease.

The role of LUS in COVID-19 patients to evaluate several respiratory conditions is nowadays widely documented [13, 20–23]. Given that characteristic sonographic features agree with CT features and appear to be related to the stage of disease [14, 20, 24] the role of follow up LUS is worth further consideration.

Even though CT scans remain the central imaging tool routine use has several obvious implications. A previous study demonstrated that pathological findings were more frequent when CT was performed later during the disease, necessitating follow-up scans with concomitant exposure to radiation [25]. In addition, CT has been found to be inferior to LUS in showing smaller peri-pulmonary lesions as well as pleural and peri-pulmonary effusion [12]. The ability of LUS to detect COVID-19 pneumonia is explained by a specific distribution of mainly peripheral and posterior lung lesions [26].

Extra strain is imposed by limited knowledge of factors indicating severe disease: single parameters like respiratory rate may not sufficiently represent real-time clinical practice [4]. This was reflected in the present study, in which we could not establish any significant relationship between sonographic features and biochemical or clinical parameters. Due to this fact we decided to concentrate our correlation of sonographic features to development of severe disease defined as need for ICU-treatment during the hospital stay.

In the present study, we performed LUS in a standardized 12 area examination. 26 out of 30 patients had at least one follow up LUS. Comparison of inter-observer variability for LUS features showed good agreement between measurements. The high percentage of patients who developed severe disease (20%) is in line with former studies [5, 6].

Compatible with previous studies [19, 20], COVID-19 features were mainly observed in posterior and lower fields in both lungs. The most frequently affected lobe was the lower left lobe, followed by the lower right lobe. Affection of upper and anterior areas was more frequently observed in patients with ICU treatment, already discussed as sign for advanced disease [27]. Patients with ICU transfer showed a tendency towards bilateral lung involvement in 100%, whereas 38,8% of the remaining had unilateral pathologies.

Increased B-lines in at least one of the 12 lung areas could be observed in all 30 patients. The high prevalence correlated with former COVID-19 studies [25] and could be found in different degrees. An irregular pleural line was discovered in 83,3% at baseline LUS with higher prevalence in follow up. That strengthens statements that increase in B-lines and pleural line irregularities seem to be a feature in the early stage [14, 23]. Subpleural consolidations were observed a little rarer at baseline, which possibly points to the already suspected later development [14, 23].

Consistent with former studies pleural effusions and larger pulmonary consolidations were uncommon [22, 23]. This can be justified by the fact that detection of these features is more frequently associated with bacterial disease [23, 25], whereas signs of interstitial viral pneumonia include rather small consolidations [23]. The presumption that larger consolidations may also suggest advanced COVID-19 pneumonia [24] is reflected in our study, where we found larger pulmonary consolidation in 50% of the patients with ICU treatment and in only 4,5% of the remaining.

The secondary end point was relationship between the change in sonographic features and development of severe disease.

The principal finding was a statistically significant association between change in cumulative B-lines and ICU-transfer. For cumulative B-lines we used a total LUS score with a maximum of 24 points. ICU-transferred patients previously developed an increase in the total LUS score in 87,5%, whereas in the remaining patients the percentage was much lower. Additionally, in all patients with ICU treatment a pattern with coalescent, bilateral B-lines could be visualized, confirming statements that an increase in B-lines matches the alveolar interstitial syndrome as main feature in the progressive stage [3, 14]. The majority of patients with a decrease in B-lines or stable results were discharged from the hospital without ICU treatment. Sensitivity and specifity of an increase in B-lines for indicating ICU transfer were 88% and 89%. The mean value of B-lines in patients with ICU transfer was higher than in the remaining patients.

Regarding the relationship between pleural line irregularities and ICU transfer a statistically significant association could be observed as well. Sensitivity and specifity of an increase in pleural line irregularities for indicating ICU transfer were 63% and 94%. Patients with stable results or decrease had to be transferred to ICU in only 15%, of those with an increase in 83%. ICU transferred patients previously developed an increase in 62,5% compared to 5,6% in the remaining patients. This fortifies the suspicion that involvement of the pleural line follows the degree of the interstitial syndrome [23].

Sensitivity and specifity of an increase in subpleural consolidations for ICU transfer were 75% and 67%. A statistically significant association could not be evidenced. Nevertheless we observed an increase in 75% in patients with ICU treatment compared to only 33,3% in the remaining patients. This confirms that progressive disease leads to an increase in subpeural consolidations in number and size [27]. We mainly observed these features in posterior and basal fields what can be explained by the fact that subpleural consolidations are associated with a discrete, localized pleural effusion [3, 22].

At time of advanced disease, a clinical characteristic is the need for supplemental oxygen [27]. Since former studies stressed the fact that oxygen requirement should trigger further evaluation [4] we evaluated oxygen demand in correlation with LUS parameters. Here we observed that increases in cumulative B-lines, subpleural consolidations and pleural line irregularities were usually associated with oxygen demand from beginning on or over time.

## Conclusions

Our study suggests that follow up LUS has major utility for management of COVID-19 patients. We hypothesize further that by observing sonographic features one can better follow the evolution of the disease. Increase in B-lines and pleural line irregularities indicated a need for ICU treatment in statistical significance. For screening routine, we recommend LUS at least on posterobasal and upper anterior fields, as pathologies were most likely to be found in the lower lobes, but affection of the upper lobe was more frequently observed in patients with ICU treatment. To our best knowledge, this is the first study investigating the relationship between change in sonographic features and clinical outcome in COVID-19 patients.

Limitation of the study is related to the small size sample and the lack of a systemic comparison with CT. Main strength is that we had a prospective design with follow up LUS, performed by the same clinician per individual patient and evaluated by experienced and blinded physicians. Additionally, the 12 area LUS provides a potential mechanism to quantify the level of involvement. However, larger studies could be necessary to provide more insight to subgroups and further strengthen the role of LUS in monitoring COVID-19 pneumonia.

## Supporting information

S1 Data (XLSX)
